# Elevated mutation rates underlie the evolution of the aquatic plant family Podostemaceae

**DOI:** 10.1038/s42003-022-03003-w

**Published:** 2022-01-20

**Authors:** Natsu Katayama, Satoshi Koi, Akira Sassa, Tetsuya Kurata, Ryoko Imaichi, Masahiro Kato, Tomoaki Nishiyama

**Affiliations:** 1 Graduate School of Science, Chiba University, Chiba, 263-8522 Japan; 2 Research Fellow of Japan Society for the Promotion of Science, Tokyo, 102-0083 Japan; 3 Botanical Gardens, Osaka City University, Osaka, 575-0004 Japan; 4 Division of Biological Science, Nara Institute of Science and Technology, Nara, 630-0192 Japan; 5 Department of Chemical and Biological Sciences, Faculty of Science, Japan Women’s University, Tokyo, 112-8681 Japan; 6 Department of Botany, National Museum of Nature and Science, Tsukuba, 305-0005 Japan; 7 Division of Integrated Omics Research, Research Center for Experimental Modeling of Human Disease, Kanazawa University, Kanazawa, 920-0934 Japan

**Keywords:** Plant evolution, Molecular evolution

## Abstract

Molecular evolutionary rates vary among lineages and influence the evolutionary process. Here, we report elevated genome-wide mutation rates in Podostemaceae, a family of aquatic plants with a unique body plan that allows members to live on submerged rocks in fast-flowing rivers. Molecular evolutionary analyses using 1640 orthologous gene groups revealed two historical increases in evolutionary rates: the first at the emergence of the family and the second at the emergence of Podostemoideae, which is the most diversified subfamily. In both branches, synonymous substitution rates were elevated, indicating higher mutation rates. On early branches, mutations were biased in favour of AT content, which is consistent with a role for ultraviolet light-induced mutation and habitat shift. In ancestors of Podostemoideae, DNA-repair genes were enriched in genes under positive selection, which may have responded to the meristem architectural changes.

## Abstract (web summary)

Katayama et al. perform transcriptome-based molecular evolutionary analyses on eight species from the aquatic plant family Podostemaceae to investigate its evolutionary history of mutation rate and genes under positive selection. Their results show that the mutation rate increased with the emergence of the family and at the emergence of the subfamily Podostemoideae, and with DNA-repair genes particularly under positive selection.

## Introduction

Evolution is affected by various factors that are unique to each taxon or species, such as ecology (environmental factors, distribution patterns, life history), development (developmental constraints), and genetic basis (mutation rate, genetic variation). In recent years, the concept of mutation as a driving force of adaptive evolution^1^ has received increasing attention, although the notion remains controversial^2^. Large-scale studies have suggested that molecular evolutionary rates correlate with species diversity and morphological evolution in both plants and animals^3–5^, while some plants show high phenotypic diversity with low genetic diversity^6^. In plants, elevated molecular evolutionary rates have been reported in carnivores and parasites^7,8^. In parasitic plants, both nonsynonymous and synonymous substitution rates increased, suggesting an elevated mutation rate^6,7^. While such plants are thought to have evolved novel morphologies under special selective pressures, an increase in genetic variation arising from elevated mutation rates is also likely to be responsible for their novel evolutionary history. It is widely accepted that the generation time is important in molecular evolution; the shorter generation time, the more mutation accumulates across generations per absolute time^9^. However, the reason behind the elevation of substitution rates in various plants is not well understood. Although rarely mentioned, environmental mutagenic pressure may also vary; for example, ultraviolet (UV) light is known as a mutagen in vitro^10^, considered to affect domestication of plants^11^, and the intensities vary among the habitat. Here, we report a higher molecular evolutionary rate in the odd aquatic plant family Podostemaceae than in a terrestrial sister group and discuss the role of elevated mutation rates in their unique evolution.

**Fig. 1:**
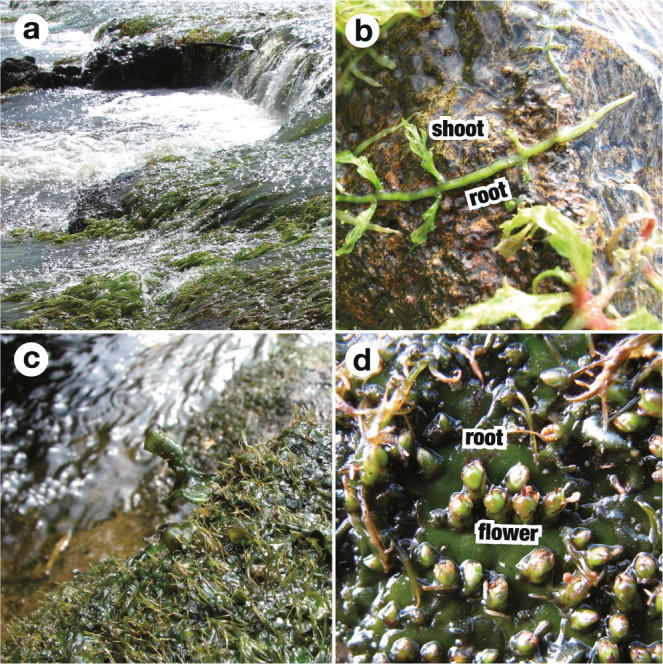
Habitats and plants of Podostemaceae. **a**–**d** Podostemad plants in natural populations. **a**, **b** *Apinagia longifolia*. **a** Plants emerging on rocks in rapids. **b** Green, ribbon-like roots creep on rock surfaces and form dissected shoots on the lateral sides. **c**, **d** *Hydrobryum japonicum*. **c** Riverbed rock covered by green foliose roots with reduced shoots scattered on the dorsal surface. **d** Flowers on foliose roots.

Podostemaceae, commonly known as river weeds, are exclusively aquatic eudicots mainly distributed in the tropics and subtropics. They grow on rocks in rapid streams and waterfalls (Fig. 1a, b). All members have a similar lifestyle, in which plants grow on submerged rocks during the rainy season. When the dry season starts and the rocks are exposed to the air, the plants enter a reproductive phase to dry out and die (Fig. 1c, d). They show unique morphologies that presumably evolved as adaptations to aquatic habitats by developmental reductionism, leading to characteristics such as loss of indeterminate shoot apical meristem (SAM) and loss of primary roots^12–17^.

Typical flowering plants grow upright with underground roots and terrestrial shoots, facilitated by indeterminate activities of the root apical meristems and SAMs. In contrast, Podostemaceae plants lack a typical root–shoot system, instead forming adventitious roots from the hypocotyls, which are green and grow on rocks. Shoots form repetitively on the roots, establishing a prostrate body plan^12,14,17^. Unusual morphological variations appear within Podostemaceae as a result of diversification in their unique habitats.

The Podostemaceae appeared relatively recently among flowering plants, after diverging from Hypericaceae ~80 Ma^18^. Podostemaceae diversified into 313 species of 53 genera, most of which are small genera of 10 or fewer species, and 42% of which (22 genera) are monotypic, reflecting rapid morphological evolution^19–22^. Podostemaceae are classified into three subfamilies, among which Trisichoideae are the most basally diverged, being sister to the monophyletic Weddellinoideae and Podostemoideae^19,23,24^. While Tristichoideae and Weddellinoideae are relatively small groups that retain a shoot system with a dome-shaped SAM, Podostemoideae evolved a unique shoot with a determinate SAM that differentiates into leaves^25^. This subfamily eventually diversified into 47 genera and 294 species.

The drastic evolution of the body plan and the high species diversity of Podostemoideae raises the possibility that the subfamily may have experienced a high molecular evolutionary rate. Longer branches of the Podostemaceae lineage compared to other taxa of Malpighiales were found in previous phylogenetic studies^24,25^. However, because these studies were conducted with limited taxon sampling in Podostemaceae (not all three subfamilies)^26,27^, or using a limited region (only plastid genome)^22,25^, historical trends of the evolutionary rate of nuclear genes among the three subfamilies have not been clarified. Therefore, analyses based on the sufficient taxon sampling and sequence information of various genes are needed for understanding the trend of evolutionary rates in this enigmatic family. In this study, we examined samples of all three subfamilies and performed high-throughput cDNA sequencing (RNA-seq) analyses to investigate when and how the evolutionary rate changed, and whether a specific set of genes experienced positive selection.

## Results

### RNA-seq and de novo assembly

**Fig. 2:**
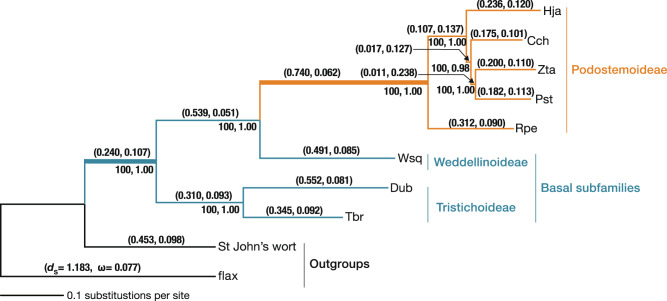
Phylogenetic tree constructed using multiple sequence alignments of 1640 orthogroups in eight Podostemads, St. John’s wort, and flax. A maximum-likelihood tree was inferred using RAxML under the GTR + Г + I model. The tree was rooted with flax. The branches of Podostemaceae are coloured blue for basal subfamilies (Tristichoideae and Weddellinoideae) and orange for Podostemoideae. Branches featured in gene ontology (GO) enrichment analysis are presented by thick lines. The horizontal lengths are proportional to the estimated number of substitutions per site under the GTR + Г + I model and bootstrap values and posterior probabilities in ASTRAL are shown below branches. The *d*_S_ and ω ratios are presented within parentheses on each branch. Cch *Cladopus chinensis*, Dub *Dalzellia ubonensis*, Hja *Hydrobryum japonicum*, Pst *Polypleurum stylosum*, Rpe *Rhyncholacis* cf. *penicillata*, Tbr *Terniopsis brevis*, Wsq *Weddellina squamulosa*, Zta *Zeylanidium tailichenoides*.

We extracted total RNA from the vegetative tissues of seven Podostemad species—two from Tristichoideae, one from Weddellinoideae, and four from Podostemoideae—and St. John’s wort (*Hypericum perforatum*) from the presumed sister clade, and performed RNA-seq analyses (Fig. 2 and Supplementary Tables 1, 2). At least 25 million paired reads were collected and assembled using Trinity^28^. The contigs were filtered to retain at least 150 amino acids and minimise redundancy (see “Methods” section). We finally obtained 28,421–78,650 contigs in each species for subsequent analyses (Supplementary Table 2).

### Acceleration of molecular evolution in Podostemaceae

**Fig. 3:**
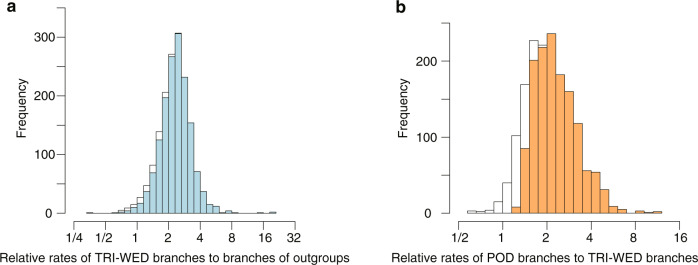
Distribution of the relative substitution rates of 1640 orthogroups. **a** Histogram of the relative substitution rates of the branches of basal Tristichoideae and Weddellinoideae compared with the outgroup branches. **b** Histogram of the relative substitution rates of Podostemoideae branches compared with the branches of basal Tristichoideae and Weddellinoideae. The distribution of the orthogroups, in which relative-rate tests showed significant results (*q* < 0.05), is coloured blue (**a**) and orange (**b**). POD Podostemoideae, TRI Tristichoideae, WED Weddellinoideae.

We inferred 1640 one-to-one orthologous gene groups (orthogroups) from contigs obtained from the RNA-seq data of seven Podostemad species, St. John’s wort, and transcript sequences obtained from the *Cladopus chinensis*^29^ and the flax (*Linum usitatissimum*) genomes^30^. Flax is the closest species for which a genome with high-quality annotation is publicly available. Using the 1640 orthogroups, we performed a phylogenetic analysis of the concatenated sequences using the maximum-likelihood method under the GTR + Γ + I model and obtained a well-supported topology (bootstrap values = 100 for all branches). ASTRAL analysis also strongly supported the same topology (posterior probabilities = 1.0 for all but one branch with posterior probability = 0.98; Supplementary Fig. 1). In the maximum-likelihood tree, longer branches were observed for Podostemaceae (Fig. 2). The tree suggests that the substitution rates increased twice within the family: first at the emergence of Podostemaceae and again at the divergence of the subfamily Podostemoideae. Discordance analysis showed that monophyly of each Podostemoideae, Tristichoideae, and the clade uniting Podostemoidea and Weddelinoideae was supported with more than 1580 genes out of 1640 and that less than 10 genes strongly rejected the monophyly of Podostemoideae, indicating a quite low discordance among the gene trees (Supplementary Table 3). We tested whether the elevated substitution rates were the result of fluctuations within finite observation or a reflection of different evolutionary rates. The following three hypotheses were compared (Table 1): H0: all branches have the same rate; H1: branches of Podostemaceae have rates different from those of St. John’s wort and flax; H2: branches of Podostemoideae have rates different from those of basal Tristichoideae and Weddellinoideae. Likelihood ratio tests were significant for H1 − H0 (2Δl = 48,901, d.f. = 1, *P* < 0.001) and H2 − H1 (2Δl = 38,248, d.f. = 1, *P* < 0.001), indicating that the preferred model is H2; that is, an elevation of substitution rates occurred at each stem branch in the family Podostemaceae and the subfamily Podostemoideae. Next, to clarify whether the elevated evolutionary rates were common among genes or specific to a given subset of genes, we performed relative-rate tests on the 1640 genes independently under the model H2. In the basal branches of the subfamilies Tristichoideae and Weddellinoideae, we found that 98.1% (1609 genes) had higher evolutionary rates than those in the branches of the outgroups (Fig. 3a), and 98.5% (1616 genes) had higher evolutionary rates in the Podostemoideae branches compared with the basal subfamilies (Fig. 3b). In both cases, the distribution of relative rates was approximately log-normal, and the peaks clearly deviated from 1 (Fig. 3; *t*-test for mean of log ratio = 0: *t* = 92.50, d.f. = 1639, *P* < 2.2e−16 and *t* = 77.03, d.f. = 1639, *P* < 2.2e−16), and the means of relative rates were 2.43 (basal subfamilies/outgroups) and 2.28 (Podostemoideae/basal subfamilies). The elevated evolutionary substitution rates, therefore, did not occur in specific genes, but widely in the genome, and the increase was more than two-fold, on average. The relative substitution rates became elevated to similar levels in both nucleotides and amino acids (mean values: 2.43 vs. 2.16 in basal subfamilies, 2.28 vs. 2.36 in Podostemoideae).

**Table 1:** Results of relative-rate test using Global and Local clock models in PAML.

Hypothesis	Model	No. of parameters	Background	Foreground	Patameter estimates (rates for branches)	Log-likelihood	AIC (−2l + 2 K)
H0	Global clock	9				−10823239.4	21646496.8
H1	Local clock	10	Outgroup	Podostemaceae	Podostemaceae: 2.57	−10798788.9	21597595.8
H2	Local clock	11	Outgroup	TRI = WED, POD	TRI + WED: 2.25 POD: 4.74	−10779665.0	21559348

*POD* Podostemoideae, *TRI* Tristichoideae, *WED* Weddellinoideae

Next, we investigated whether the mutation rates were elevated because the mutation is a major driver of molecular evolution. While nonsynonymous substitutions (*d*_N_) can change protein functions and are subject to natural selection, synonymous substitutions often have weak selective effects and are expected to behave as nearly neutral mutations^4,31^. Differences in the synonymous substitution rate (*d*_S_) are, therefore, mainly attributable to differences in the underlying mutation rate^32^. In the *d*_S_ tree using concatenated sequences, there were long branches in the family Podostemaceae. On average, the substitution rates in the basal branches of Podostemaceae were 2.40 times greater than those of the outgroups, and the rates in the Podostemoideae branches were 1.70 times those of the basal subfamilies (Fig. 2). These results suggest that spontaneous mutation rates in this family increased, resulting in accelerated molecular evolution.

### Investigating the causal factors for the acceleration of mutation rates

**Fig. 4:**
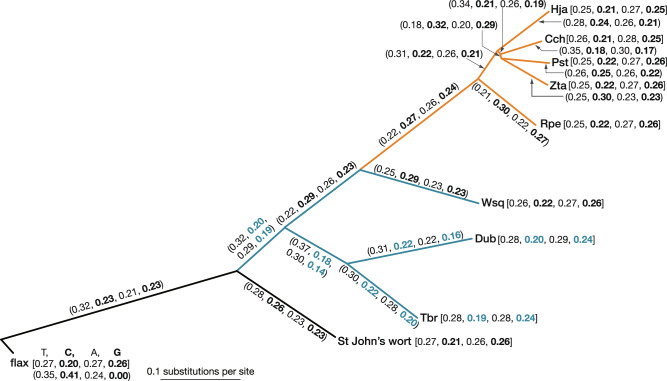
A maximum-likelihood tree and estimates of base-frequency parameters for branches using a concatenated alignment of 1640 orthogroups. Numbers in parentheses are estimates of the frequency parameters in the HKY + dG+N2 model for the branch, while those in brackets are the base frequencies at the nodes. The frequency parameters and the base frequencies are shown in the order T, C, A, G in parentheses and brackets. Cch *Cladopus chinensis*, Dub *Dalzellia ubonensis*, Hja *Hydrobryum japonicum*, Pst *Polypleurum stylosum*, Rpe *Rhyncholacis* cf. *penicillata*, Tbr *Terniopsis brevis*, Wsq *Weddellina squamulosa*, Zta *Zeylanidium tailichenoides*. Bar indicates 0.1 substitutions per site.

A given mutagen or specific mutational process tends to produce specific types of mutation, giving rise to a mutation spectrum. For example, UV light preferentially induces C-to-T mutations (G-to-A in the complementary DNA strand)^10,11^. If the composition of a mutagen changed during evolution, the substitution process would be non-stationary and the base frequency would also change. To infer the history of the mutational spectrum trend, we used a model in which the equilibrium frequencies differed among branches^33^. The observed frequencies of C and G were lower than 0.25 in Tristichoideae (*T. brevis* and *D. ubonensis*; Fig. 4), and the estimated equilibrium frequency of C and G leading to those branches and the ancestral branch of Podostemaceae was low (0.14–0.22), particularly in the branch uniting the Tristichoideae (C: 0.18, G: 0.14; Fig. 4). This observation is consistent with the hypothesis that UV-induced mutations increased at the emergence of Podostemaceae.

To further investigate evidence as to the causes of the increased evolutionary rate in this family, we attempted to detect positive selection at the stem branch of Podostemaceae. We used a branch-site model to detect positive selection affecting specific sites along particular lineages^34–36^ and investigated the functions of the positively-selected genes using gene ontology (GO) enrichment analysis. Since many sites were tested, some of the sites found in this analysis may be false positives. However, significant GO enrichment is unlikely to occur because of random false positives, and is therefore likely to reflect evolutionary forces. To increase the size of the gene set to be analysed, incomplete orthogroups lacking one Asian Podostemoideae species were also added to the analyses. Of a total of 1964 orthogroups, 459 genes had at least one site under positive selection with a posterior probability above 95%, according to a Bayes Empirical Bayes (BEB) method^36^ (Supplementary Data 1). We used GO annotations of the flax gene to represent an orthogroup for the enrichment analysis. Among the 1964 orthogroups, GO annotations were assigned to 1150 orthogroups (Supplementary Data 2). GO enrichment analysis revealed no significant enrichment in the Biological Processes, but response to light stimulus popped at a high rank with complete retainment (Supplementary Data 3). Two genes were retained; one is *PHYTOCHROME C* (*PHYC*) and the other is *CPSRP43* encoding chloroplast signal recognition particle 43 (cpSRP43). Although neither protein is involved in UV resistance, PHYC functions as a light receptor for photoperiodic flowering^37,38^ and cpSRP43 helps plants to adjust their chlorophyll synthesis rate in response to environmental change^39^.

There were no visible spectrum changes in GC and AT frequencies at the Weddellinoideae and Podostemoideae branches. This finding implies that factors other than UV promoted the elevation of molecular evolutionary rates at the Podostemoideae branches. To characterise the evolutionary process during the second elevation of mutation rates for the subfamily Podostemoideae, we attempted GO enrichment analysis for the positively-selected genes at the stem branch of Podostemoideae as well. In the stem branch for Podostemoideae, 511 genes had experienced positive selection on at least one site (Supplementary Data 4). Of the 511 genes, 238 GO annotations were assigned and GO enrichment analysis revealed enrichment of GO:0006281 “DNA repair” (20/829 to 10/238, *P* = 0.0342) (Supplementary Data 5). Seven other genes involved in DNA damage response were also found in the list of the 511 positively-selected genes (Supplementary Data 4).

**Fig. 5:**
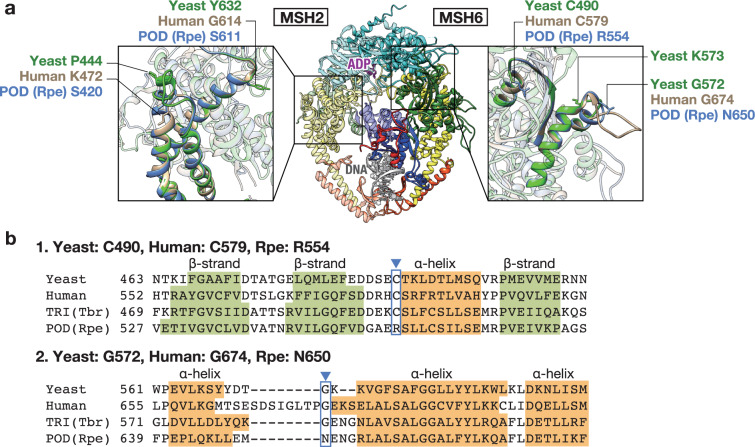
Prediction of Podostemaceae MSH2/MSH6 complex structures. **a** Superimposed three-dimensional models of yeast and Podostemaceae MSH2 (left) and MSH6 (right) proteins with human MutSα (MSH2/MSH6) bound to ADP and DNA strands (PBD ID: 2O8B). Red: disordered domain, green: connector domain, blue: mismatch binding domain, yellow: lever domain, orange: clamp domain, light blue: ATPase domain. The inset figures show magnifications of the regions around the positively-selected sites. The structure of the *Rhyncholacis* cf. *penicillata* (Podostemaceae) MSH2/MSH6 is shown in blue, with human MSH2/MSH6 in silver, and yeast MSH2/MSH6 in green. **b** MSH6 alignment of the regions around the first and second positively-selected sites (arrowheads). Predicted secondary structures are shown. POD Podostemoideae, Rpe *Rhyncholacis* cf. *penicillata*, Tbr *Terniopsis brevis*, TRI Tristichoideae.

Of the 10 genes associated with “DNA repair,” we found a photolyase that repairs UV-induced DNA damage^40^ and five genes involved in major DNA-repair mechanisms: MutS homologue 2 (MSH2) and MSH6 act in the DNA-mismatch repair system, DNA-(apurinic or apyrimidinic site) lyase and N-glycosylase/DNA lyase (OGG1) in the base-excision repair, and general transcription factor IIH subunit 2 in the nucleotide excision repair^40^. The base-excision repair and nucleotide excision repair pathways suppress UV-induced mutagenesis by directly removing DNA photoproducts and oxidative lesions formed by UV irradiation^41^. Mismatch repair also plays a key role in genome integrity by correcting the mismatched nucleotides that occur during DNA synthesis in UV-induced DNA damage^42^. In these six genes, the positively-selected sites were located in conserved domains (Supplementary Data 6–11). Positively-selected sites were detected in both MSH6 and MSH2, which form a heterodimer and act in mismatch repair (Fig. 5a). Their function is conserved in bacteria (*MutS*) and eukaryotes^43,44^. We found two positively-selected sites in the connector domain of MSH6 (Fig. 5a, b, full alignment in Supplementary Data 6). One was a highly conserved site in budding yeast, humans, *Arabidopsis*, Tristichoideae, and Weddellinoideae, but it is changed in the Podostemoideae species. The other site was also well conserved from yeast to the basal subfamilies of Podostemaceae, and a previous complementation study in yeast showed that a mutant allele *msh6*-573 (K573D K574D), with substitutions next to that site (G572) failed to fully complement the mutator phenotype of a Δ*msh3* Δ*msh6* strain^45^. A surface loop 650–675 in human MSH6 is one of three loops proposed to mediate protein–protein interactions^44^. Yeast MSH6p interacts with NHP6Ap, an HMGB1 homologue, and modulates their loading to DNA substrate^46^. An HMGB1-interacting sequence corresponding to amino acids 631–637 in human MSH6 has been recognised, but not experimentally identified^43^. These amino acid changes in the connector domain could influence the stability of the genome and the efficacy of DNA repair. In MSH2, two positively-selected sites were found, both of which were located in the lever domain, which functions in opening the cavity to which DNA binds, by conformational change with the clamp domain^47^. Missense mutations at the positively-selected sites detected in this study have been reported to be “functionally neutral,” indicating that missense mutations are deleterious in MSH2 knockout human cells^48^.

**Fig. 6:**
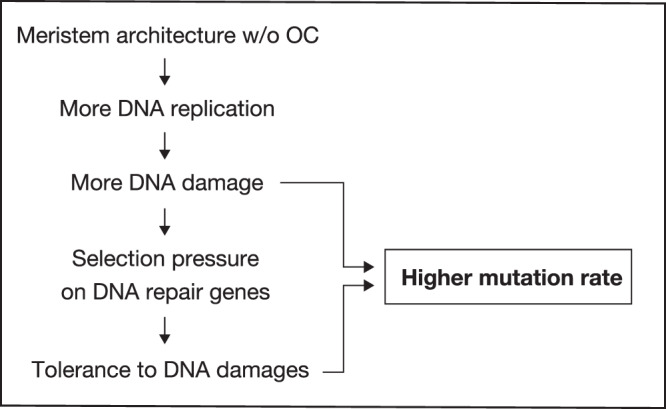
Hypothetical scenario leading to higher mutation rates in Podostemoideae. In Podostemoideae, the meristem architecture was changed to one without the organising centre (OC). The change led to more DNA replication in the generation and more DNA damage, which could have caused higher mutation rates. Further, more DNA damage would result in more selection pressure on DNA repair genes, such that more DNA damages are tolerated. The tolerance to DNA damages again could lead to higher mutation rates through survival of the cells with DNA damage leading to mutations rather than cell death.

Modification of the meristem maintenance system occurred in Podostemoideae. Although GO enrichment analysis did not detect terms related to shoot development, we identified nine genes that may be involved in the regulation of shoot meristem activity and development (Supplementary Data 4). The ephemeral fate of the shoot meristems leads to the accumulation of somatic mutations throughout the lives of the plants. DNA repair may have increased the tolerance to DNA damage, allowing cells with damaged DNA to proliferate (Fig. 6). Cells with a deficiency in mismatch-repair capacity are reportedly more tolerant to specific types of DNA damages^49^.

### Relaxed selection in the Podostemoideae crown groups

**Fig. 7:**
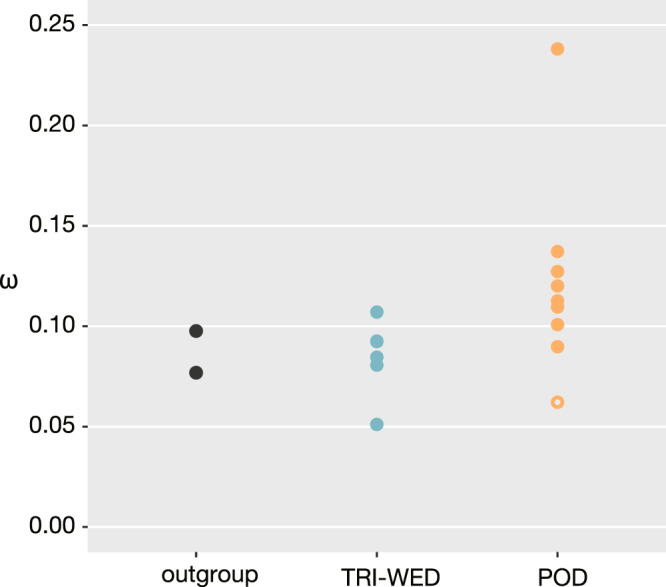
Plot of ω in the branches of outgroup, basal subfamilies, and Podostemoideae. The orange open circle indicates the Podostemoideae stem branch. POD Podostemoideae, TRI Tristichoideae, WED Weddellinoideae.

The increase in the ω ratio is a signal of relaxed functional constraints^50,51^. We examined the ω of the branches using a concatenated alignment under a free-ratio model^52,53^. Higher ω values were obtained from the Podostemoideae branches (0.090–0.238) than from the branches of the basal subfamilies (0.051–0.107) and the Podostemoideae stem branch (0.062) (Fig. 7). This observation suggests that the constraints relaxed in Podostemoideae, which experienced drastic morphological changes. The colonisation of new habitats usually occurs with a reduction in effective population size^54,55^, which in turn leads to lower selection pressure. Population fragmentation can also reduce the effective population size. Podostemaceae species usually consists of small populations separated by a number of rapids and waterfalls^22^, making migration between locations difficult. Relaxation of constraints through radiation and fragmented population structure may have contributed to the accelerated accumulation of nonsynonymous changes in the Podostemoideae crown group.

## Discussion

Our results demonstrate that the rate of molecular evolution in the aquatic plant family Podostemaceae accelerated twice. In both cases, the acceleration can be attributed primarily to elevated mutation rates, accompanied by the relaxation of selection pressures in the second acceleration.

The plants of the family Podostemaceae colonised rocks in rivers upon which the sunlight falls directly. We found a trend toward lower GC frequencies in the basal branches of Podostemaceae, which indicates a UV-induced mutational spectrum. GO enrichment analysis indicated probable positive selection on *PHYC* and *CPSRP43*, which act in response to light stimuli, supporting the connection that the light environment of the habitat might have changed at the time of emergence of the family. The light energy from various types of light, including UV light and ionising radiation, causes DNA damage both directly and through the generation of reactive oxygen species^56,57^. Excess visible light can also lead to the production of reactive oxygen species during the process of photosynthesis^58^. These results are consistent with a scenario in which light-induced mutations increased along with habitat changes at an early evolutionary stage. Because members of Podostemaceae have an annual lifestyle adapted to seasonal water level changes, they are particularly exposed to sunlight in the open areas of rivers at the beginning and the end of their life cycles. The generation time of the annual lifestyle is also one of the factors leading to higher mutation rates, coupled with habitat changes at the beginning of the evolutions of the family^27^.

At the time of the emergence of the subfamily Podostemoideae, the evolutionary rates accelerated further, and positive selection on DNA-repair genes was detected. We also identified several genes related to shoot and root development that might be under positive selection. Although GO enrichment analysis did not show significant enrichment of these genes, they could have been involved in the evolution of shoot architecture. One likely explanation is that the acceleration of mutation rates in Podostemoideae is a consequence of the modification of the meristem structure, which is unique to Podostemoideae. In the centre of the SAM of most flowering plants, there is a small group of cells, known as the organising centre (OC), which divide at a slower rate than the surrounding cells and maintain the cell fate^59–61^. Because cell division involves DNA replication, it can be a mutation source^62,63^. Somatic mutations in the SAM that are transmitted to the next generation could be restricted to low levels by minimising the number of cell divisions that occur in the OC^64,65^. In contrast, in Podostemoideae, the loss of the ordinary maintenance system of the shoot meristem via the OC during architectural change^25^ could increase the number of somatic mutations transmitted to the next generation (Fig. 6). Meristematic cells that are not under the control of the OC experience more DNA replication and cell division cycles, increasing the number of mutations occurring, with the same replication accuracy. Because DNA might be more susceptible to damage during replication, selection pressure to tolerate DNA damage could have been strengthened, leaving traces in DNA-repair genes. Some forms of mismatch-repair deficiency reportedly increase tolerance to specific types of DNA damage^49^, implying the existence of a trade-off between tolerance to DNA damage and mutation rate. DNA-repair mechanism may, therefore, allow cells with damaged DNA to proliferate at the cost of a higher mutation rate (Fig. 6).

Our results were slightly different from those of previous studies^24,27^. Phylogenetic analyses using the *matK* sequences of 132 species by Koi et al.^24^ and the plastid genomes of five species investigated by Bedoya et al.^27^ showed longer branches in Tristichoideae than in Podostemoideae. Both Koi et al.^24^ and Bedoya et al.^27^ analysed plastid genes, while our analyses were of nuclear genes. Plastids and nuclear DNA reside in different cellular compartments. The DNA-repair genes that we found as having positively-selected mutations in Podostemoideae are probably localised in the nucleus, but not in plastids. Thus, plastid and nuclear DNA evolution may have involved different forces in different lineages.

While the heterogeneity of molecular evolutionary rates is widely known, the way in which the rate of molecular evolution affects the evolutionary process remains an open question because the interactions are complex and can vary among taxa. Despite the limited number of species included in the present study, we detected general trends in the elevation of molecular evolutionary rates at the subfamily level in Podostemaceae. The causes of accelerated molecular evolution were investigated using transcriptome-based molecular evolutionary analyses involving a large number of one-to-one orthologous groups. We predicted that changes in the light environment at the time of emergence of the family, and the modification of the stem cell maintenance and DNA-repair systems during the divergence of Podostemoideae, are probable causes of the accelerated substitution rates. Experimental measurements of mutation rates in organisms from environments with a range of natural UV level variations are currently unavailable, and should be considered as future work. The details of the effects of the mutations that are likely to have been affected by the positive selection should also be the subject of future studies. As high-throughput transcriptome sequencing of multiple closely related taxa is now feasible, similar analyses should reveal evolutionary aspects in other taxa with accelerated evolutionary rates that have experienced drastic developmental and physiological evolution, such as parasites and carnivores. The establishment of a general rule for changes in the rate of molecular evolution requires a better understanding of the mechanisms behind various cases.

## Methods

### Plant materials and culture conditions

Seeds of Podostemaceae species (*T. brevis*, *D. ubonensis*, *W. squamulosa*, *R*. cf. *penicillata*., *Z. tailichenoides*, *P. stylosum*) and plant bodies of *H. japonicum* were collected from wild populations (Supplementary Table 1). Voucher specimens were deposited in the Herbarium of the National Museum of Nature and Science, Japan (TNS). Seeds of St. John’s wort (*H. perforatum* L. ‘Helos’) were purchased from Richters Herbs (Goodwood, Ontario, Canada). For seedling culture, Podostemad seeds were placed on 3.0% agar containing 0.05% (v/v) HYPONeX liquid fertiliser (Major nutrients: N-P-K = 6-10-5; Hyponex Japan, Tokyo, Japan) and the agar medium was covered with 0.05% (v/v) HYPONeX liquid medium. The plants were cultured in a growth chamber at 25 °C under a long-day light regime (14 h light/10 h dark). St. John’s wort seeds were cultured on 0.8% agar medium at 25 °C under a light regime of 16 h light/8 h dark.

### RNA extraction and RNA sequencing

For RNA-seq, we used seedlings of Podostemaceae and St. John’s wort 1–30 days after sowing, including shoot and root organs but not reproductive organs. *H. japonicum* plants with roots, shoots, and flowers were collected in the field and soaked in RNA*later* (ThermoFisher Scientific, Waltham, MA, USA) solution at the site. The samples were frozen in liquid nitrogen and crushed with zirconia beads. Total RNA was extracted using PureLink Plant RNA Reagent (ThermoFisher Scientific) and treated with TURBO DNase (ThermoFisher Scientific). To construct the RNA-seq libraries, we used several construction kits and performed Illumina paired-end sequencing (Supplementary Table 2). The raw read data were deposited in the DDBJ Sequence Read Archive (DRA) under the accession numbers DRA008126 and DRA010548–DRA010554.

### De novo assembly and construction of one-to-one orthogroups

We constructed one-to-one orthogroups following the methods described in Amemiya^3^. Reads from each species were assembled using Trinity^28^. The transcripts were translated, and only the longest open reading frame, of at least 150 amino acids, was retained for each transcript. Redundancy was minimised using the dereplication mode of USEARCH v6.0.307. Groups of orthologous proteins were determined using SonicParanoid, leading to the identification of 6723 orthogroups, each of which included at least one sequence for each of the 10 species. In the second step, the 6723 orthogroups were aligned with MAFFT version 7^66^ using the E-INS-i strategy. One representative sequence per species was selected for each orthogroup using SCaFoS^67^. Only orthogroups without missing species were retained, yielding a data set of 1796 orthogroups. To exclude potentially non-homologous sequence segments, we used HmmCleaner^68^. Ambiguously aligned positions were removed using Gblocks^69^ with default parameter settings. After these filtration steps, sequences with at least 100 amino acids were retained. The matrices were converted again to nucleotides, with the final data set consisting of 1,739,691 nucleotide positions in 1640 orthogroups.

We also made orthogroups that allow one missing species from Asian Podostemoideae clades consisting of *Z. tailichenoides, P. stylosum, H. japonicum* and *C. chinensis*, to increase the power of detection of positive selection. In SCaFoS, we obtained 76 one-to-one orthogroups without *Z. tailichenoides*, 53 without *P. stylosum*, 164 without *H. japonicum* and 70 without *C. chinensis*, resulting in a total of 363 orthogroups. After HmmCleaner, Gblocks and size filtering were applied to for these orthogroups, 324 orthogroups were retained.

### Phylogenetic analysis of multi-gene sequences

RAxML 8.0.5 was used to construct a maximum-likelihood tree under the GTR + Γ + I model using all 1,739,691 positions of the 1640 orthogroups. Bootstrap values were calculated for 10,000 replicates. Further, we inferred a coalescent-based species tree from 1640 gene trees using ASTRAL-III^70^ and measured branch supports as local posterior probabilities^71^. Discordance analysis on gene trees was performed using DiscoVista^72^ and branches with bootstrap support values above (below) 75% were considered to be highly (weakly) supported. A clade that is not in the original tree but is compatible if low support branches (below 75%) were contracted is considered as a weakly rejected clade. A clade that is not compatible even after contracting low support branches is regarded as strongly rejected.

### Analyses of molecular evolutionary rates and positive selection

Molecular evolutionary analyses were performed using the PAML4 programme package^73^. To perform likelihood ratio tests among the hypotheses under different evolutionary models (Table 1), we obtained the log-likelihood values of H0 (the global clock model), H1 (the local clock model), and H2 (the local clock model) using the codeml programme and performed chi-squared tests. In relative-rate tests of each of the 1640 orthogroups, we obtained *P*-values from the chi-squared tests of the log-likelihood under H0 vs. H1, and H1 vs. H2 and adjusted false discovery rate *q*-values using the fdrtool package in R v3.5.1^74^. Relative rates were obtained under model H2.

To estimate the change in the number of synonymous substitutions, we constructed a *d*_S_ tree of 1640 orthogroups using a free-ratio branch model implemented in the codeml programme. We calculated the average branch lengths of each of the following groups: outgroups, basal subfamilies, and Podostemoideae. In the concatenated sequences, estimates of nucleotide frequency parameters for the branches and the base frequencies were obtained using the baseml programme under the HKY + dG+N2 model^33^. To detect genes that experienced positive selection at the stem branch of the family Podostemaceae and the subfamily Podostemoideae, we applied a branch-site model to the sequences of each of the 1964 orthogroups including those with no missing species and those with one missing species. Positively-selected sites (*ω* > 1) were identified using BEB methods for calculating the posterior probabilities for the site classes (class 0: negative, class 1: neutral, class 2a and 2b: positive). Estimates of the ω ratio on the branches were also obtained using concatenated alignments of 1640 orthogroups under a free-ratio branch model to infer the relaxed constraints in each branch.

### Gene ontology enrichment analysis

GO enrichment analysis was carried out with a Cytoscape plugin BiNGO^75^ using annotation data of flax (*Linum usitatissimum* v1.0 in Phytozome).

### MSH2/MSH6 3D structure prediction

Protein structure homology-modelling of yeast and Podostemaceae MSH6 and MSH2 was performed using the SWISS-MODEL programme (https://swissmodel.expasy.org/)^76^. Human MutSα (MSH2/MSH6) complex (PDB ID: 2O8B) was used as a template to generate the homology models, which were visualised using Chimera 1.11.2^77^.

### Reporting summary

Further information on research design is available in the Nature Research Reporting Summary linked to this article.

## Supplementary information

Supplementary Information

Description of Additional Supplementary Files

Supplementary Data 1

Supplementary Data 2

Supplementary Data 3

Supplementary Data 4

Supplementary Data 5

Supplementary Data 6

Supplementary Data 7

Supplementary Data 8

Supplementary Data 9

Supplementary Data 10

Supplementary Data 11

Supplementary Data 12

Supplementary Data 13

Supplementary Data 14

Reporting Summary

## Data Availability

Source data underlying figures are presented in Supplementary Data 12–14. The raw read data of RNA-seq are deposited in DRA (DDBJ Read Archive) and available through DRA/SRA/ENA with the accession numbers (DRR238797–DRR238827, DRR258784-DRR258790; Supplementary Table 2). The assembled contig sequences are deposited in the TSA division of DDBJ (INSDC accession ICRX01000001–ICSE01072389, Supplementary Table 2) with notes on the orthogroup assigned. The input data and outputs of molecular evolutionary analyses are deposited in Dryad (10.5061/dryad.z34tmpgft)^78^.
