# Supplementary material for: Elevated mutation rates underlie the evolution of the aquatic plant family Podostemaceae
Source: Commun Biol. 2022 Jan 20;5:75. doi: 10.1038/s42003-022-03003-w (PMC8776956; doi:10.1038/s42003-022-03003-w)
Supplement: Supplementary file 1 — Supplementary Information [file 42003_2022_3003_MOESM1_ESM.pdf]

Supplementary Table 1. List of material information used in this study.

Family	Species	Locality	Voucher
Linaceae	Flax (Linum usitatissimum)	-	-
Clusiaceae	St. John's wort (*Hypericum perforatum*)	Richters Herbs (Goodwood, Ontario, Canada).	-
Podostemaceae	*Terniopsis brevis*	Kaeng Lamduan stream, Yoddome Wildlife Sanctuary, Ubon Ratchathani, Thailand	TNS-8200205
Podostemaceae	*Dalzellia ubonensis*	Lamduan stream, Yod Dome wildlife sancturary, Ubon Ratchathani, Thailand	TIK-30
Podostemaceae	*Weddellina squamulosa*	Goat Falls, Essequibo River, Kurupukari, Guyana	TNS-8200203
Podostemaceae	*Rhyncholacis* cf. *penicillata*	Papikai Falls, Mazaruni River, Shanklands, Guyana	TNS-8000127
Podostemaceae	*Polypleurum stylosum*	Panathur, Kasaragod District, Kerala, Kerala, India	TNS-8200119
Podostemaceae	*Zeylanidium tailichenoides*	Huay Kaew stream, Maethakhrai National Park, Chiang Mai, Thailand	TNS-8000188
Podostemaceae	*Cladopus chinensis*	-	-
Podostemaceae	*Hydrobryum japonicum*	Interior to the Ogawa Electric Power Station, Nejime, Minami-Osumi, Kimotsuki, Kagoshima.	TNS-761724

Supplementary Table 2. List of nucleotide sequence information used in this study.

Species	Data	Library preparation Kit	Platform	Read format	Number of runs (biological rep, technical rep)	Number of total reads (M reads)	Assembly software	Number of contigs after assembly	Number of contigs after filtering	DDBJ accession Number (DRA/TSA)
Flax (*Linum usitatissimum*)	CDS sequences (Linum usitatissimum v1.0) obtained from Phytozome 12	-	-	-	-	-	-	-	43484 transcripts	-
St. John's wort (*Hypericum perforatum*)	RNA-seq	TruSeq Stranded mRNA sample preparation kit (Illumina)	Illumina HiSeq1500	PE:101bp	8 (4, 2)	199.7	Trinity v. v2.0.6	243833	59093	DRR238810-DRR238817/ ICRZ01000001-ICRZ01243830
Terniopsis brevis	RNA-seq	TruSeq Stranded mRNA sample preparation kit (Illumina)	Illumina HiSeq1500	PE:101bp	4 (2, 2)	197.6	Trinity v. v2.0.6	94619	31262	DRR238822-DRR238825/ ICSD01000001-ICSD01094581
Dalzellia ubonensis	RNA-seq	TruSeq Stranded mRNA sample preparation kit (Illumina)	Illumina HiSeq2500/1500	PE:101bp	6 (3, 2)	111.7	Trinity v. v2.0.6	79109	31472	DRR238797-DRR238802/ ICRX01000001-ICRX01079073
Weddellina squamulosa	RNA-seq	TruSeq Stranded mRNA sample preparation kit (Illumina)	Illumina HiSeq2500	PE:101bp	2 (2, 1)	39	Trinity v. r20140717	72393	33530	DRR238826-DRR238827/ ICSE01000001-ICSE01072389
Rhyncholacis cf. *penicillata*	RNA-seq	TruSeq Stranded mRNA sample preparation kit (Illumina)	Illumina HiSeq2500	PE:101bp	2 (2, 1)	25.3	Trinity v. r20140717	58260	29045	DRR238820-DRR238821/ ICSC01000001-ICSC01058253
Polypleurum stylosum	RNA-seq	TruSeq Stranded mRNA sample preparation kit (Illumina)	Illumina HiSeq2500	PE:101bp	2 (2, 1)	34.2	Trinity v. r20140717	62281	28412	DRR238818-DRR238819/ ICSB01000001-ICSB01062274
Zeylianidium tailichenoides	RNA-seq	RNA-Seq Library Construction Kit (Ambion), Next mRNA Sample Prep Reagent Set 1 (NEB), SMARTer cDNA Synthesis Kit (Clontech)	Illumina GA IIx	PE:100bp	7 (1, 7)	238.4	Trinity v. r2012-01-25	100357	31173	DRR258784-DRR258790/ ICSA01000001-ICSA01100340
Cladopus chinensis	CDS sequences obtained from the BIG Sub system under BioProject accession number CRA002215.	-	-	-	-	-	-	-	27270	-
Hydrobryum japonicum	RNA-seq	TruSeq Stranded mRNA sample preparation kit (Illumina)	Illumina HiSeq2500	PE:101bp	7 (7, 1)	131.9	Trinity v. r20131110	338312	78650	DRR238803-DRR238809/ ICRY01000001-ICRY01338312

Supplementary Table 3. Results of discordance analysis on 1640 gene trees using DiscoVista.

CLADE	Strongly Supported	Weakly Supported	Weakly Reject	Strongly Reject
Podostemoideae	1622	11	6	1
Tristichoideae	1536	72	26	6
Weddellinoideae/Podostemoideae	1501	85	46	8
Tristichoideae/Weddellinoideae	5	19	110	1506

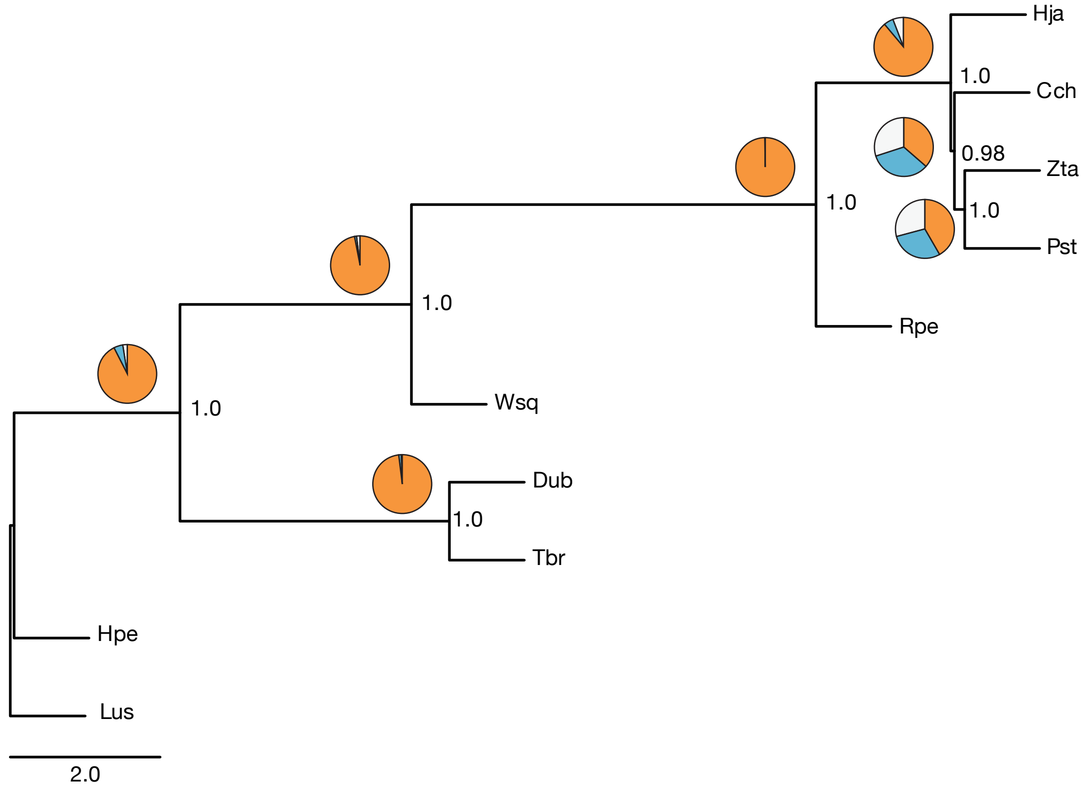

Supplementary Figure 1.
Species tree inferred by ASTRAL-III based on 1,640 gene trees. The tree was rooted at *Linum usitatissimum*. Numbers are local posterior support. ASTRAL-III measures branch length in coalescent units in internal nodes. Pie charts show relatiuve frequency of the three quartet topologies in all the gene trees that support the main topology (Orange), the first alternative (blue), and the second alternative (white). Cch: *Cladopus chinensis*, Dub: *Dalzellia ubonensis*, Hja: *Hydrobryum japonicum*, Hpe: *Hypericum perforatum*, Lus: *Linum usitatissimum*, Pst: *Polypleurum stylosum*, Rpe: *Rhyncholacis* cf. *penicillata*, Tbr; *Terniopsis brevis*, Wsq: *Weddellina squamulosa*, Zta; *Zeylanidium tailichenoides*.
