# Supplementary material for: Elevated mutation rates underlie the evolution of the aquatic plant family Podostemaceae
Source: Commun Biol. 2022 Jan 20;5:75. doi: 10.1038/s42003-022-03003-w (PMC8776956; doi:10.1038/s42003-022-03003-w)
Supplement: Supplementary file 2 — Description of Additional Supplementary Files [file 42003_2022_3003_MOESM2_ESM.pdf]

Description of Additional Supplementary Files

File name: Supplementary Data 1
Description: Annotation list of 459 positively-selected genes detected at the stem branch of Podostemaceae.

File name: Supplementary Data 2
Description: List of orthologous groups with GO ID used in GO enrichment analysis.

File name: Supplementary Data 3
Description: List of GO enrichment analysis for positively-selected genes at the stem branch of Podostemaceae.

File name: Supplementary Data 4
Description: Annotation list of 511 positively-selected genes detected at the stem branch of the subfamily Podostemoideae.

File name: Supplementary Data 5
Description: List of GO enrichment analysis for positively-selected genes at the stem branch of the subfamily Podostemoideae.

File name: Supplementary Data 6
Description: Multiple sequence alignment of DNA mismatch repair protein MSH6 and alignment positions of positively selected sites.

File name: Supplementary Data 7
Description: Multiple sequence alignment of DNA mismatch repair protein MSH2 and alignment positions of positively selected sites.

File name: Supplementary Data 8
Description: Multiple sequence alignment of DNA-(APURINIC OR APYRIMIDINIC SITE) LYASE (APEX) and alignment positions of positively selected sites.

File name: Supplementary Data 9
Description: Multiple sequence alignment of transcription initiation factor TFIIH subunit 2 (TF2H2) and alignment positions of positively selected sites.

File name: Supplementary Data 10
Description: Multiple sequence alignment of N-glycosylase/DNA lyase (OGG1) and alignment positions of positively selected sites.

File name: Supplementary Data 11
Description: Multiple sequence alignment of deoxyribodipyrimidine photo-lyase (PHR) and alignment positions of positively selected sites.

File name: Supplementary Data 12
Description: Source data for Figs. 2, 4.

File name: Supplementary Data 13
Description: Source data for Fig. 3.

File name: Supplementary Data 14
Description: Source data for Fig. 6.
