# Supplementary material for: Elevated mutation rates underlie the evolution of the aquatic plant family Podostemaceae
Source: Commun Biol. 2022 Jan 20;5:75. doi: 10.1038/s42003-022-03003-w (PMC8776956; doi:10.1038/s42003-022-03003-w)
Supplement: Supplementary file 17 — Reporting Summary [file 42003_2022_3003_MOESM17_ESM.pdf]

nature research

Corresponding author(s): Natsu Katayama, Tomoaki Nishiyama

Last updated by author(s): Dec 1, 2021

Reporting Summary

Nature Research wishes to improve the reproducibility of the work that we publish. This form provides structure for consistency and transparency in reporting. For further information on Nature Research policies, see our Editorial Policies and the Editorial Policy Checklist.

Statistics

For all statistical analyses, confirm that the following items are present in the figure legend, table legend, main text, or Methods section.

n/a	Confirmed	
☐	☒	The exact sample size (n) for each experimental group/condition, given as a discrete number and unit of measurement
☒	☐	A statement on whether measurements were taken from distinct samples or whether the same sample was measured repeatedly
☐	☒	The statistical test(s) used AND whether they are one- or two-sided<br>*Only common tests should be described solely by name; describe more complex techniques in the Methods section.*
☒	☐	A description of all covariates tested
☐	☒	A description of any assumptions or corrections, such as tests of normality and adjustment for multiple comparisons
☐	☒	A full description of the statistical parameters including central tendency (e.g. means) or other basic estimates (e.g. regression coefficient) AND variation (e.g. standard deviation) or associated estimates of uncertainty (e.g. confidence intervals)
☐	☒	For null hypothesis testing, the test statistic (e.g. F, t, r) with confidence intervals, effect sizes, degrees of freedom and P value noted<br>*Give P values as exact values whenever suitable.*
☒	☐	For Bayesian analysis, information on the choice of priors and Markov chain Monte Carlo settings
☒	☐	For hierarchical and complex designs, identification of the appropriate level for tests and full reporting of outcomes
☒	☐	Estimates of effect sizes (e.g. Cohen's d, Pearson's r), indicating how they were calculated

Our web collection on statistics for biologists contains articles on many of the points above.

Software and code

Policy information about availability of computer code

Data collection	Standard softwares accompanying the sequencers were used.
Data analysis	All of softwares used in this study are present in a main text or Methods section.

For manuscripts utilizing custom algorithms or software that are central to the research but not yet described in published literature, software must be made available to editors and reviewers. We strongly encourage code deposition in a community repository (e.g. GitHub). See the Nature Research guidelines for submitting code & software for further information.

Data

Policy information about availability of data

All manuscripts must include a data availability statement. This statement should provide the following information, where applicable:

- Accession codes, unique identifiers, or web links for publicly available datasets
- A list of figures that have associated raw data
- A description of any restrictions on data availability

Source data underlying figures are presented in Supplementary Data 12–14. The raw read data of RNA-seq are deposited in DRA (DDBJ Read Archive) and available through DRA/SRA/ENA with the accession numbers (DRR238797–DRR238827, DRR258784–DRR258790; Supplementary Table 2). The assembled contig sequences are deposited in the TSA division of DDBJ (Supplementary Table 2) with notes on the orthogroup assigned. The input data and outputs of molecular evolutionary analyses are deposited in Dryad (https://doi.org/10.5061/dryad.z34tmpgft) 75.

Field-specific reporting

Please select the one below that is the best fit for your research. If you are not sure, read the appropriate sections before making your selection.

☐ Life sciences ☐ Behavioural & social sciences ☒ Ecological, evolutionary & environmental sciences

For a reference copy of the document with all sections, see nature.com/documents/nr-reporting-summary-flat.pdf

Ecological, evolutionary & environmental sciences study design

All studies must disclose on these points even when the disclosure is negative.

Study description	We designed the study to investigate whether the molecular evolutionary rates of Podostemaceae is faster than that of closely related taxa and infer what kinds of factors associated with the major evolutionary events of Podostemaceae.
Research sample	We used eight species of Podostemaceae, a sister group of Hypericum perforatum to be compared with Podostemaceae, and a Linum usitatissimum that belongs to the same Malpighiales as an outgroup and has available genomic information.
Sampling strategy	Sampling of Podostemaceae plants was performed widely from all three subfamilies so that comparison of molecular evolutionary rates could be performed within the family.
Data collection	We collected total RNA from seedlings except for Hydrobryum japonicum that grow in Japan because cultivation of Podostemaecae plants is difficult in lab. Since we could not obtain seeds, total RNA of H. japonicum was obtained from adult plants.
Timing and spatial scale	2005-02-23 to 2013-10-09; once and one locality for each species.
Data exclusions	No data were excluded.
Reproducibility	Analysis were made on assembled contigs that represents multiple sequence reads. All results in this study can be reproduced from the data provided as Supplementary data files.
Randomization	The analyses were based on phylogenetical relationship.
Blinding	Nucleotide sequence are called by computer programs accompanied with the sequencer and no human change is made.

Did the study involve field work? ☐ Yes ☒ No

Reporting for specific materials, systems and methods

We require information from authors about some types of materials, experimental systems and methods used in many studies. Here, indicate whether each material, system or method listed is relevant to your study. If you are not sure if a list item applies to your research, read the appropriate section before selecting a response.

Materials & experimental systems

n/a	Involved in the study	
☒	☐	Antibodies
☒	☐	Eukaryotic cell lines
☒	☐	Palaeontology and archaeology
☒	☐	Animals and other organisms
☒	☐	Human research participants
☒	☐	Clinical data
☒	☐	Dual use research of concern

Methods

n/a	Involved in the study	
☒	☐	ChIP-seq
☒	☐	Flow cytometry
☒	☐	MRI-based neuroimaging
